# Wild mushrooms as potential reservoirs of plant pathogenic bacteria: a case study on *Burkholderia gladioli*

**DOI:** 10.1128/spectrum.03395-23

**Published:** 2024-02-21

**Authors:** Mozhde Hamidizade, S. Mohsen Taghavi, Ardavan Soleimani, Mohammad Bouazar, Hamid Abachi, Perrine Portier, Ebrahim Osdaghi

**Affiliations:** 1 Department of Plant Protection, School of Agriculture, Shiraz University, Shiraz, Iran; 2 Department of Plant Protection, College of Agriculture, University of Tehran, Karaj, Iran; 3 Univ Angers, Institut Agro, INRAE, IRHS, SFR QUASAV, CIRM-CFBP, Angers, France; 4 Center for International Scientific Studies and Collaborations (CISSC) of Iran, Tehran, Iran; Pennsylvania State University, University Park, Pennsylvania, USA

**Keywords:** clinical bacteria, cross-kingdom pathogen, edible mushroom, soft rot bacteria, *Suillus luteus*

## Abstract

Fruit bodies (sporocarps) of wild mushrooms growing in natural environments play a substantial role in the preservation of microbial communities, for example, clinical and food-poisoning bacteria. However, the role of wild mushrooms as natural reservoirs of plant pathogenic bacteria remains almost entirely unknown. Furthermore, bacterial transmission from a mushroom species to agricultural plants has rarely been recorded in the literature. In September 2021, a creamy-white Gram-negative bacterial strain was isolated from the sporocarp of *Suillus luteus* (slippery jack) growing in Bermuda grass (*Cynodon dactylon*) lawn in Southern Iran. A similar strain was isolated from the same fungus in the same area in September 2022. Both strains were identified as *Burkholderia gladioli* based on phenotypic features as well as phylogeny of 16S rRNA and three housekeeping genes. The strains were not only pathogenic on white button mushrooms (*Agaricus bisporus*) but also induced hypersensitive reaction (HR) on tobacco and common bean leaves and caused soft rot on a set of diverse plant species, that is, chili pepper, common bean pod, cucumber, eggplant, garlic, gladiolus, narcissus, onion, potato, spring onion, okra, kohlrabi, mango, and watermelon. Isolation of plant pathogenic *B. gladioli* strains from sporocarp of *S. luteus* in two consecutive years in the same area could be indicative of the role of this fungus in the preservation of the bacterium in the natural environment. *B. gladioli* associated with naturally growing *S. luteus* could potentially invade neighboring agricultural crops, for example, vegetables and ornamentals. The potential role of wild mushrooms as natural reservoirs of phytopathogenic bacteria is further discussed.

**IMPORTANCE:**

The bacterial genus *Burkholderia* contains biologically heterogeneous strains that can be isolated from diverse habitats, that is, soil, water, diseased plant material, and clinical specimens. In this study, two Gram-negative pectinolytic bacterial strains were isolated from the sporocarps of *Suillus luteus* in September 2021 and 2022. Molecular phylogenetic analyses revealed that both strains belonged to the complex species *Burkholderia gladioli*, while the pathovar status of the strains remained undetermined. Biological investigations accomplished with pathogenicity and host range assays showed that *B. gladioli* strains isolated from *S. luteus* in two consecutive years were pathogenic on a set of diverse plant species ranging from ornamentals to both monocotyledonous and dicotyledonous vegetables. Thus, *B. gladioli* could be considered an infectious pathogen capable of being transmitted from wild mushrooms to annual crops. Our results raise a hypothesis that wild mushrooms could be considered as potential reservoirs for phytopathogenic *B. gladioli*.

## INTRODUCTION

Deciphering the survival modes of plant pathogenic bacteria in the absence of their main host plant plays a pivotal role in understanding the corresponding disease cycle (1). Most ecological studies on the survival of bacterial phytopathogens are focused on the role of seeds, plant debris, soil, non-host plants, weeds, and machinery in this phenomenon (2–4). Depending on the fundamental biological features of each bacterial pathogen, one or some of these habitats act as a natural reservoir of primary inoculum for the establishment of the disease. Within the past few decades, there has been an increasing interest in elucidating the role of non-agronomic biological niches in the survival of plant pathogenic bacteria (5). For instance, Morris et al. (6) showed that the life cycle of phytopathogenic *Pseudomonas syringae* includes a wide range of natural reservoirs, for example, rain, snow, alpine streams, and lakes as well as wild plants and epilithic biofilms. Recently, microbiome studies based on the metagenomics of non-agronomic environments revealed that a number of plant pathogenic bacteria—or their very close relatives—were present in lichens (symbiotic associations of fungi, algae, and bacteria) (7, 8). While plant pathogenicity has not been tested for most of these microbial communities associated with environmental vegetation, Vilhelmsson et al. (5) noted that these new habitats could be considered potential reservoirs for established or emerging plant diseases. All this evidence raised the question of whether the fungal constituents of agronomic environments, for example, sporocarps of wild mushrooms, could act as reservoirs of plant pathogenic bacteria. On the other hand, the potential transmission of an infectious bacterium from a sporocarpous fungus to an agricultural plant remains unclear.

From 2018 to 2022, we have conducted a series of comprehensive surveys to monitor the microbial communities associated with edible and wild mushrooms in Iran. These mushrooms were either produced under a protected environment or grown under natural conditions (9–11). Samplings from the natural environments were conducted year-round and repeated for two years to provide an inclusive insight into the microbial dynamics of sporocarps of wild mushrooms in each area. In this framework, apart from dozens of environmental strains, that is, enterobacteria, pseudomonads, and bacilli (11, 12), a Gram-negative bacterial strain (named Ir1503) was isolated from the sporocarp of the wild mushroom *Suillus luteus* (slippery jack) naturally grown in Bermuda grass (*Cynodon dactylon*) lawn in Shiraz county in September 2021. In the subsequent year (September 2022), another strain (Ir1504) similar to that isolated in 2021 was isolated from the sporocarps of the same mushroom (*S. luteus*) in the same location. Preliminary investigations, that is, colony color, morphology, and pigmentation suggested that both strains belonged to *Burkholderia* sp. (13).

Plant pathogenic members of *Burkholderia* include *Burkholderia cepacia*, *Burkholderia cenocepacia*, two pathovars of *Burkholderia gladioli* (i.e., *B. gladioli* pv. *gladioli* and *B. gladioli* pv. *alliicola*), and *Burkholderia glumae*. Furthermore, *B. gladioli* pv. *agaricicola* causes soft rot of edible mushrooms (14). *B. glumae* is pathogenic on rice [causing panicle blight (15)], while *B. cepacia*, *B. cenocepacia*, and *B. gladioli* induce soft rot diseases on a set of taxonomically diverse vegetables, ornamentals, and mushrooms (16–19). Since the beginning of the current century, classification of *Burkholderia* spp. has undergone various changes both in the species and infra-species levels. Pathogenicity and host range of the species have also been studied on various hosts (20–22). For instance, Jones et al. (23) analyzed genome sequences of 206 *B. gladioli* strains from diverse origins, showing that *B. gladioli* pv. *alliicola* and food-poisoning *B. gladioli* pv. *cocovenenans* strains were distinct, while *B. gladioli* pv. *gladioli* and *B. gladioli* pv. *agaricicola* were indistinguishable based on genomic and phylogenomic analyses. Moreover, it has been shown that soft-rot activity is a universal feature across *B. gladioli* lineages where pathovars of the species have overlapping host ranges and the pathotype strains could not be distinguished based on experimental host range assay (23).

Preliminary *in vitro* screenings showed that the two bacterial strains isolated from *S. luteus* in this study were capable of inducing soft rot on fleshy plant tissues. These observations raised a hypothesis that the wild mushroom *S. luteus* could be a potential reservoir of plant pathogenic bacteria, for example, *Burkholderia* sp. under natural conditions. Thus, the purpose of this study was to investigate the biological characteristics, pathogenicity, and host range, as well as taxonomic status of the *Burkholderia* strains isolated from *S. luteus*. Furthermore, we performed a series of biological assays to see if mushroom-associated *Burkholderia* strains could act as a potential plant pathogen under experimental conditions.

## RESULTS

### Bacterial strains

The Gram-negative bacterial strains Ir1503 and Ir1504 were isolated from brown-colored sporocarps of *S. luteus* in September 2021 and September 2022, respectively. The sporocarps of *S. luteus* had brown pits while no tissue maceration and malformation were observed. The two bacterial strains had circular creamy-white colonies with yellowish-green non-fluorescent pigmentation on the agar medium, with smooth (Ir1503) and wrinkled (Ir1504) margins, and were 1 to 2 mm in diameter. The two bacterial strains were oxidase-negative, catalase-positive, and obligate aerobic. The strains were positive in urease production, Tween 80 hydrolysis, and utilization of D-sorbitol, raffinose, inositol, and D-mannitol. They also had pectinase and protease activity, hydrolyzed casein, and induced hypersensitive reaction (HR) on tobacco (*Nicotiana tabacum* cv. Turkish) and common bean leaves (Fig. S1A through C). However, they were negative in amylolytic activity and levan production. Based on these phenotypic characteristics, the two strains were preliminarily identified as members of *Burkholderia* spp.

### Pathogenicity on edible mushroom

**Fig 1:**
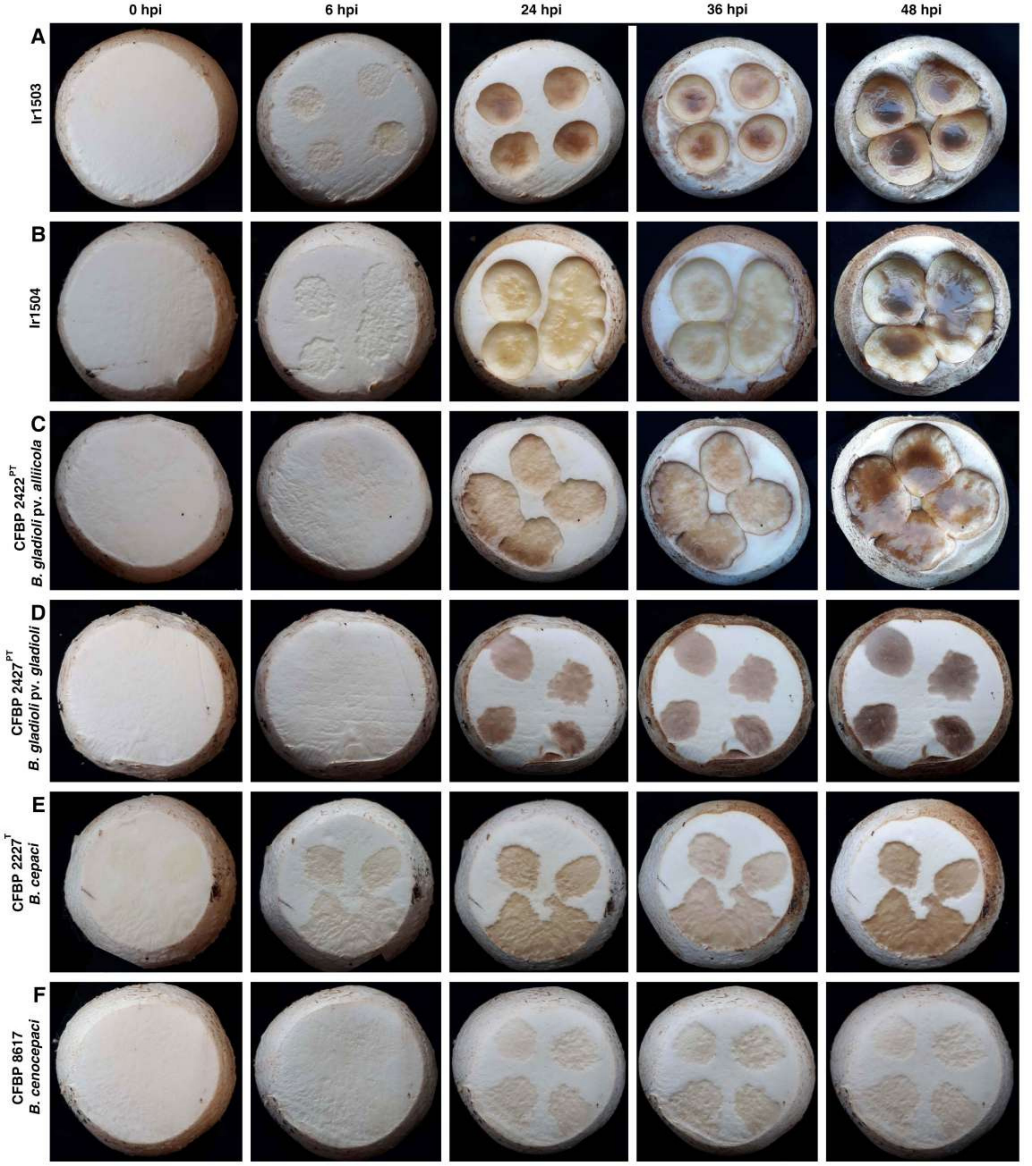
Pathogenicity of *B. gladioli* strains isolated from the sporocarp of *S. luteus* on *Agaricus bisporus* cap under controlled conditions. The strains Ir1503 (**A**) and Ir1504 (**B**) as well as the pathotype strain of *B. gladioli* pv. *alliicola* CFBP 2422^PT^ (**C**) induced severe pitting and soft rot on the sporocarp of button mushrooms. However, the pathotype strain of *B. gladioli* pv. *gladioli* (CFBP 2427^PT^, **D**) and the type strain of *B. cepacia* (CFBP 2227^T^, **E**) induced only brown blotch on the mushroom caps, while *B. cenocepacia* CFBP 8617 (**F**) had only a slight effect on the cap. hpi, Hours post inoculation.

Both strains Ir1503 and Ir1504 induced brown pitting and tissue rot on button mushroom caps 24 hours post inoculation (hpi) while initial symptoms on the specimens inoculated with both strains started as soon as 6 hpi (Fig. 1A and B). Severe soft rot and brown cavities were observed on mushroom caps inoculated with both Ir1503 and Ir1504 36–48 hpi (Fig. 1A and B). Surprisingly, the pathotype strain of *B. gladioli* pv. *alliicola* (CFBP 2422^PT^; Fig. 1C) also induced soft rot symptoms similar to those caused by the strains Ir1503 and Ir1504. Initial symptoms of CFBP 2422^PT^ started on 12 hpi and extended to the fleshy tissues making pitted slippery cavities on the cap. Pathotype strain of *B. gladioli* pv. *gladioli* (CFBP 2427^PT^), however, induced brown discoloration and blotch symptoms on mushroom caps with no tissue maceration and pitting even 48 hpi (Fig. 1D). As for type strain of *B. cepacia* CFBP 2227^T^ (Fig. 1E), inoculation of mushroom caps by CFBP 2227^T^ resulted in discoloration of the inoculated areas even 6 hpi and continued to become deep brown and slightly pitted. Considering the colony color and pigmentation of the strain CFBP 2227^T^ which is yellowish green with non-fluorescent pigmentation on the agar medium, the discoloration noted at 6 hpi for CFBP 2227^T^ could be the inoculum itself. Minimal disease symptoms were induced on the mushroom caps inoculated with *B. cenocepacia* strain CFBP 8617 where only faint creamy discoloration was observed on the specimens with no pitting, malformation, and soft rot (Fig. 1F). While brown blotch symptoms were observed on the mushroom caps inoculated with *Pseudomonas tolaasii* CFBP 8707 (positive control), the caps inoculated with non-pathogenic strain of *Escherichia coli* dh5α and sterile distilled water (SDW) remained symptomless until 72 hpi (Fig. S2).

### Plant pathogenicity and host range

**Fig 2:**
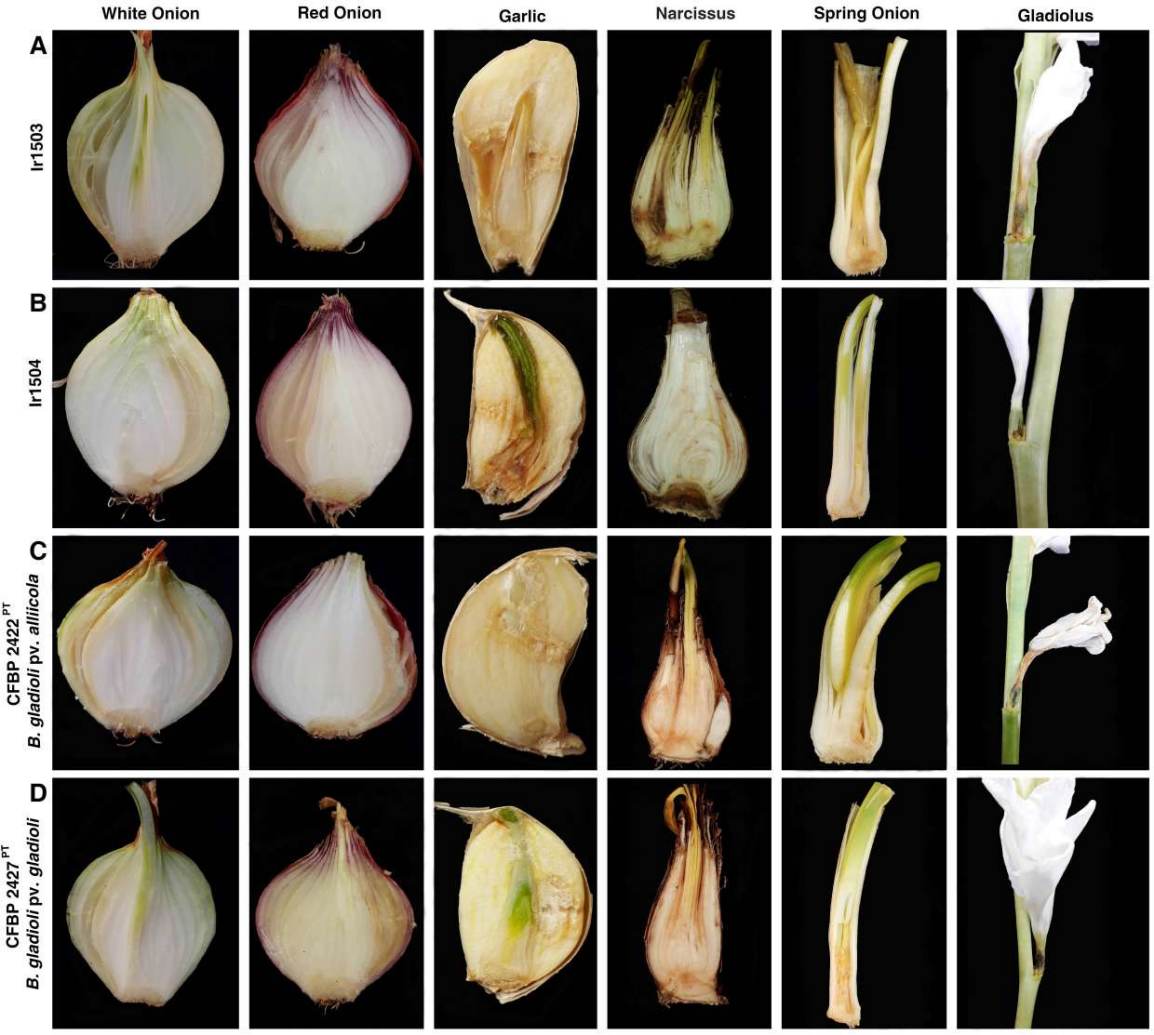
Plant pathogenicity of *B. gladioli* strains Ir1503 (**A**) and Ir1504 (**B**) isolated from the sporocarp of *S. luteus* compared to the pathotype strains of *B. gladioli* pv. *alliicola* (CFBP 2422^PT^, **C**) and *B. gladioli* pv. *gladioli* (CFBP 2427^PT^, **D**). All strains induced soft rot and tissue maceration on onion (the main host of *B. gladioli* pv. *alliicola*), garlic, spring onion, and gladiolus (main host of *B. gladioli* pv. *gladioli*). Except for the strain Ir1503 which induced black discoloration on the narcissus bulb, the other strains had a slight effect (faint discoloration) on this plant.

The strain Ir1503 isolated from *S. luteus* in 2021 was pathogenic on onion (both white and red onions) as well as taxonomically related species, that is, garlic, narcissus, and spring onion (Fig. 2A). In all plant species inoculated with the latter strain, water-soaked areas and tissue maceration were observed in the site of inoculation. In onions and spring onions, infected tissues turned to gray or light brown, while an unpleasant smell was associated with severe infections. In garlic and narcissus, infected tissues turned brown and dark brown, respectively, as shown in Fig. 2A. Brown rot discoloration and maceration were observed on the ovary of gladiolus flowers. The strain Ir1504 which was isolated in the subsequent year (September 2022) from the same fungus in the same location showed a similar virulence scheme on onions, garlic, narcissus, spring onion, and gladiolus as shown in Fig. 2B. However, the severity of the symptoms on narcissus, spring onion, and gladiolus was less than that observed on the plant’s inoculation with the strain Ir1503. Interestingly, pathotype strains of *B. gladioli* pv. *alliicola* (CFBP 2422P^T^; Fig. 2C) and *B. gladioli* pv. *gladioli* (CFBP 2427^PT^; Fig. 2D) induced soft rot and maceration on all tested plant species including the main host of each other, confirming that the pathovar designation within *B. gladioli* is not supported by experimental host range assays. None of the strains could induce soft rot on Persian shallot bulbs.

**Fig 3:**
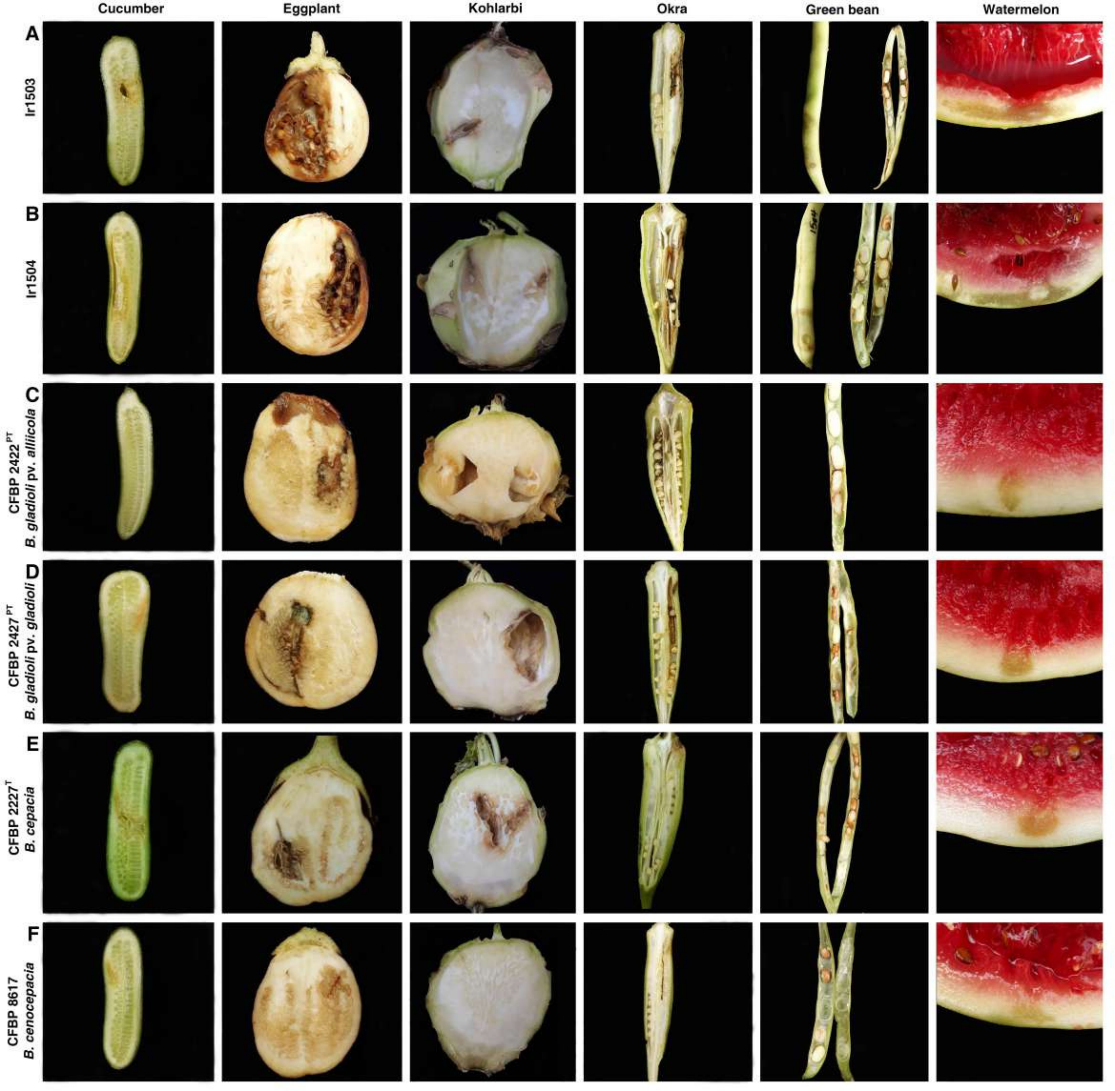
*In vitro* host range assay on cucumber, eggplant, kohlrabi, okra, green bean, and watermelon inoculated with Ir1503 (**A**) and Ir1504 (**B**) as well as the pathotype strain of *B. gladioli* pv. *alliicola* CFBP 2422^PT^ (**C**), pathotype strain of *B. gladioli* pv. *gladioli* CFBP 2427^PT^ (**D**), type strain of *B. cepacia* CFBP 2227^T^ (**E**), and *B. cenocepacia* CFBP 8617 (**F**). All strains induced soft rot and tissue maceration on cucumber, eggplant, green bean pods, kohlrabi, okra, and watermelon fruit except for *B. cenocepacia* strain CFBP 8617 which had the most restricted host range where none of the inoculated plant species showed severe soft rot and maceration.

In order to further investigate the host range and plant pathogenicity of the mushroom-associated strains beyond monocotyledonous plants, they were inoculated on a set of dicotyledonous vegetables and fruits. Both strains Ir1503 and Ir1504 induced soft rot and tissue maceration on chili pepper, cucumber, eggplant, green bean pods, kohlrabi, okra, and watermelon fruit as shown in Fig. 3A and B; Fig. S3. Brown discoloration of the inoculated tissues and cavity was observed in cucumber, eggplant, and kohlrabi. On okra and green bean pods, soft rot and discoloration were observed on green immature seeds besides the fleshy tissues of the pod. In watermelon, soft rot was observed on the rind (inoculation site), which then extended into the fleshy pulp 48 hpi, leading to the destruction of the entire fruit. Similar symptoms were observed when *B. gladioli* pv. *alliicola* CFBP 2422^PT^ (Fig. 3C), *B. gladioli* pv. *gladioli* CFBP 2427^PT^ (Fig. 3D), and *B. cepacia* CFBP 2227^T^ (Fig. 3E) were inoculated on eggplant, kohlrabi, and green bean. Pathotype strain of *B. gladioli* pv. *alliicola* did not induce soft rot on the cucumber while *B. gladioli* pv. *gladioli* and *B. cenocepacia* caused faint discoloration on the site of inoculation. The type strain of *B. cepacia* induced soft rot and cavity on cucumber similar to that observed in the specimens inoculated with Ir1503 and Ir1504. On the other hand, pathotype strains of *B. gladioli* pv. *alliicola* and *B. gladioli* pv. *gladioli* induced discoloration and soft rot on okra pods while the type strain of *B. cepacia* had no effect on this plant species. On watermelon, symptoms caused by the strains Ir1503 and Ir1504 were more severe compared to those induced by the reference strains of *B. gladioli*, *B. cepacia*, and *B. cenocepacia* as shown in Fig. 3F. Extended rind rot and discoloration followed by destruction of pulp tissues were observed on the watermelon fruits inoculated by the former strains, while symptoms caused by the latter strains were limited to water-soaked area and faint discoloration in the site of inoculation. Overall, among the strains investigated in this study, *B. cenocepacia* strain CFBP 8617 had the narrowest host range where none of the inoculated plant species showed severe soft rot and maceration. On the other hand, the strains Ir1503 and Ir1504 isolated from the wild mushroom *S. luteus* were the most aggressive strains where they induced soft rot on all plant species (except for Persian shallot) tested in this study.

### Phylogenetic analyses

**Fig 4:**
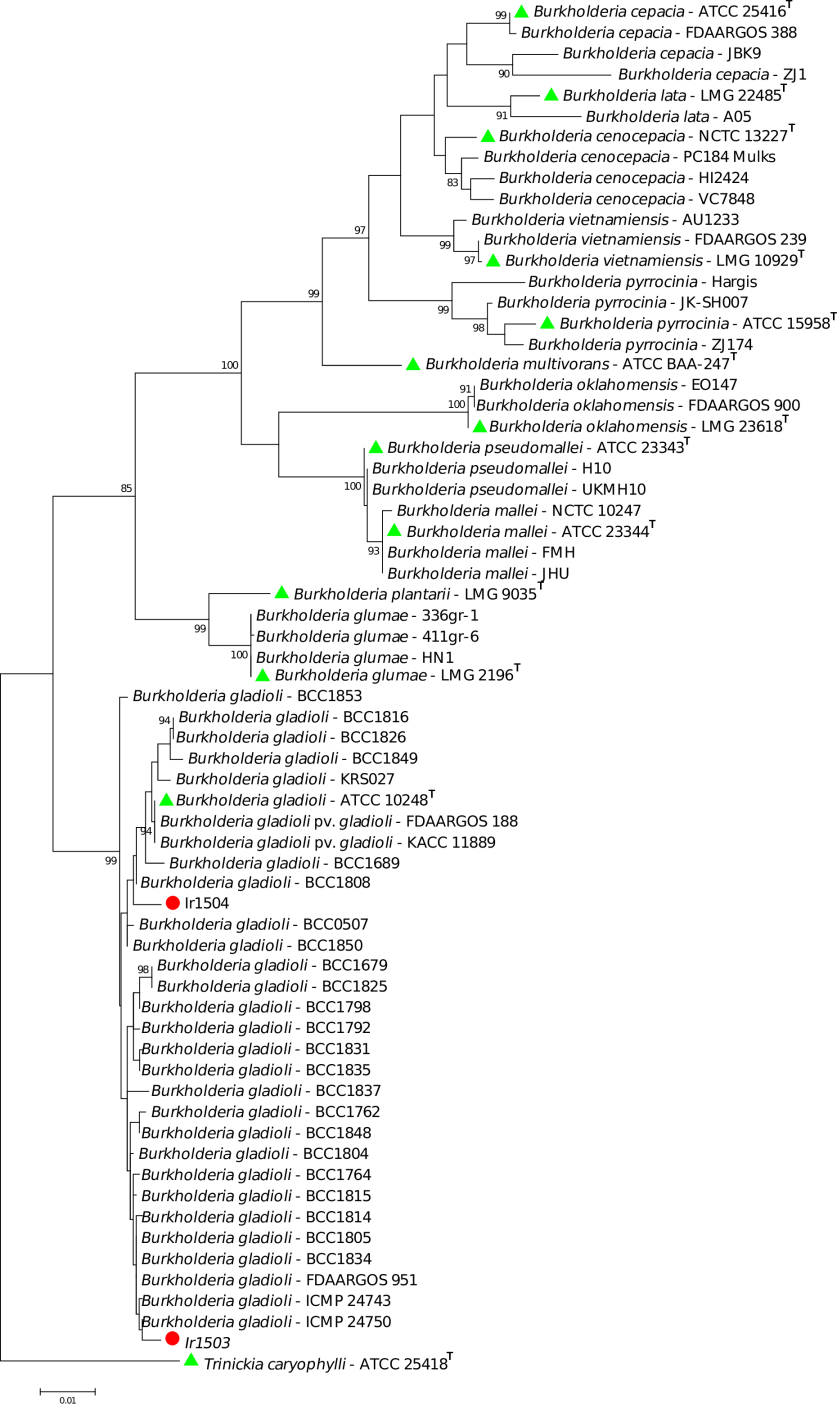
Phylogeny of *B. gladioli* strains isolated from the sporocarp of *S. luteus* among the type/standard strains of *Burkholderia* spp. based on the concatenated sequences of *atpD*, *gyrB*, and *lepA* genes. Phylogenetic analyses were performed using MEGA7 software via the Maximum Likelihood method. The strains Ir1503 and Ir1504 were identified as *B. gladioli* while Ir1504 was placed in the clade where the type strain of the species was clustered. The strain Ir1503 was clustered in another sub-clade next to the environmental and garlic pathogenic strains of the species. *Trinickia caryophylli* ATCC 25,418T (formerly known as *Burkholderia caryophylli*) was used as an out-group in the phylogenetic tree. Green triangles indicate the type strains of each species, while red circles show the strains isolated in this study.

BLAST search using the sequences of individual genes (i.e., 16S rRNA, *atpD*, *gyrB*, and *lepA*) suggested that the bacterial strains isolated in this study belong to the complex species *B. gladioli* (Fig. 4). Phylogenetic analyses using the concatenated sequences of three housekeeping genes showed that the strain Ir1503 was clustered in a sub-clade next to the environmental and garlic pathogenic strains of the species. However, the strain Ir1504 was placed in another sub-clade along with the type strain of the species (ATCC 10248^T^) as well as gladiolus-pathogenic strains of *B. gladioli* pv. *gladioli* FDAARGOS188 and KACC 11889 (Fig. 4).

## DISCUSSION

In this study, a Gram-negative pectinolytic bacterial strain was isolated from the sporocarps of *S. luteus* in September 2021. In the subsequent year, surveys were conducted in the same location on the same fungus which resulted in the isolation of another Gram-negative pectinolytic strain in September 2022 (strains Ir1503 and Ir1504, respectively). Molecular phylogenetic analyses revealed that both strains belonged to the complex species *B. gladioli* while pathovar status of the strains remained undetermined. Biological investigations accomplished with pathogenicity and host range assays showed that *B. gladioli* strains isolated from *S. luteus* in two consecutive years were pathogenic on a set of diverse plant species ranging from monocotyledonous vegetables and ornamentals, that is, onions and gladiolus to dicotyledonous annual crops, that is, chili pepper, cucumber, eggplant, green bean, kohlrabi, okra, mango, and watermelon. These results raise a hypothesis that wild mushrooms could serve as potential reservoirs for phytopathogenic *B. gladioli*. However, more directed efforts are needed to justify this conclusion.

In 1991, *Pseudomonas gladioli* pv. *agaricicola* causing bacterial soft rot of *Agaricus bitorquis* was described by Lincoln et al. (16), which was later reclassified as *B. gladioli* pv. *agaricicola* (24). The latter pathogen has rarely been investigated for its host range and biological characteristics compared with the other two pathovars in the species (23). *B. gladioli* strains isolated from *S. luteus* were aggressively pathogenic on button mushrooms, while the symptoms were indistinguishable from those induced by the pathotype strain of *B. gladioli* pv. *alliicola* CFBP 2422^PT^. Inclusion of the pathotype strains of *B. gladioli* pv. *alliicola* and *B. gladioli* pv. *gladioli* in the pathogenicity assays deciphered a mixed host range of phytopathogenic and mycopathogenic *B. gladioli* strains. Recently, we have isolated *B. gladioli* strains from garlic bulbs possessing soft rot symptoms. The garlic strains showed a wide host range including bulbous monocotyledons, dicotyledonous vegetables, and cacti, as well as wild and edible mushrooms (25). On the other hand, *B. gladioli* strains from distinct environmental and clinical sources have been reported to be pathogenic on mushrooms (23). These indications suggest that the assignment of plant-pathogenic and mushroom-pathogenic *B. gladioli* strains into different pathovars will be misleading—if not impossible due to their mixed host range—in terms of pathogen identification and disease management.

Within the past few decades, several plant pathologists have focused their studies on the role of non-agronomic biological niches in the survival of plant pathogens. For instance, Vilhelmsson et al. (5) noted that phytopathogenic bacteria including *P. syringae*, *Burkholderia glathei* [now known as *Paraburkholderia glathei* (26)], and *Xanthomonas* spp. were present in significant numbers in or on lichen thalli. A number of bacterial taxa were retrieved frequently in different lichen species sampled in the same or different sites. *Paenibacillus* sp. and *Burkholderia* sp. seem to be common in lichens (27). Furthermore, Bartoli et al. (28) noted that environmental strains of *P. syringae* were capable of causing symptoms on kiwifruit and showed a wide host range revealing their potential as future pathogens of a variety of hosts. Some strains of *B. cepacia* are adapted to the ecological niche of the rhizosphere capable of fixing nitrogen or producing indole-3-acetic acid hormone (29). *Burkholderia* spp. could survive in non-agronomic niches even in the presence of hazardous materials (30). In the present study, host range assays showed that the *B. gladioli* strains isolated from *S. luteus* are invasive pathogens of fleshy fruits, vegetables, and edible mushrooms. While the dissemination and spread of *B. gladioli* from the sporocarps of *S. luteus* to agricultural crops still remains unknown, precautions need to be taken to protect the infestation of agricultural areas with this pathogen.

In another perspective, mushroom microflora plays a pivotal role in both crop production and public health. The probability of wild mushrooms to become contaminated with plant pathogens and clinical bacterial pathogens is relatively greater than that of cultivated varieties. Contamination of wild mushrooms with bacterial pathogens can occur directly or indirectly via animals or insects. Venturini et al. (31) showed that mushrooms carry clinical bacterial species *Listeria monocytogenes* and *Yersinia enterocolitica* in Spain. In the UK, mushrooms purchased from supermarkets, originally grown in five different countries, were all positive for the presence of clinical bacterial species *Pseudomonas aeruginosa*. The number of Colony-forming unit (CFU) per gram in the outer layer of the caps was much higher than those in the whole mushrooms. In Zaragoza (Spain), 22 species of cultivated and wild fresh mushrooms sold in retail markets and supermarkets were studied by Venturini et al. (31) to quantify their microbial load. The most prevalent microbial groups included *Enterobacteriaceae*, pseudomonads, and lactic acid bacteria (31). In a study on golden chanterelle mushroom (*Cantharellus cibarius*), fluorescent pseudomonads represented 78% of the microbial load (31, 32).

The genus *Burkholderia* comprises versatile bacterial pathogens that cause severe diseases in humans (33), animals (34), and plants (35). Genetic distinctions between plant- and human-pathogenic *Burkholderia* strains are not clear (36). *B. cepacia* causes fatal pulmonary infections in cystic fibrosis patients (37). Pneumonia infections caused by *B. gladioli* and *B. glumae* in patients with chronic granulomatous disease were reported (38, 39). Septicemia caused by *B. cenocepacia* in cystic fibrosis patients was also reported (36). A number of *Burkholderia* species, that is, *B. cepacia*, *B. cenocepacia,* and *B. gladioli* are common pathogens of both animals and plants. Yet, little is known about the molecular basis of the infection, their spatial distribution, and the biological role of toxic agents involved in their virulence (40). We used reference strains of the latter three species in our biological investigations alongside our strains isolated in this study and noted that they were not only pathogenic on a diverse set of plant species but also could invasively infect edible mushrooms. All these facts highlight on the one hand the necessity of substantial investigations in the ecology of cross-kingdom *Burkholderia* species. On the other hand, reliable, efficient, and low-cost microbiological methods need to be developed for the detection, diagnosis, and differentiation of human-, plant-, and mushroom-pathogenic *Burkholderia* strains on a commercial scale.

## MATERIALS AND METHODS

### Sampling and bacterial isolation

Samplings from naturally grown wild mushrooms were conducted in September 2021 and at the same time in the subsequent year in Shiraz, Iran. Fully-grown sporocarps of wild mushrooms either grown in soil or in association with trees and shrubs were sampled and immediately transferred to the laboratory for further analyses. Microbial isolation was conducted on yeast-extract peptone glucose agar (YPGA) medium via multiple streaking as recommended by Schaad et al. (13). In brief, pieces of sporocarps were aseptically cut and macerated in a few drops of SDW using a sterile mortar and a pestle. A loopful of the resulting suspensions was streaked on YPGA medium and the plates were incubated at 27°C for 3–4 days. The resulting purified bacterial strains were re-suspended in SDW and stored at 4°C for further use or maintained in 15% glycerol at −70°C for long-term storage.

In order to determine whether the mushroom-associated bacterial strains possess phytopathogenic features, all bacterial strains were initially evaluated for pectinolytic activity and induction of HR. The HR test was conducted on tobacco (*N. tabacum* cv. Turkish) and common bean (*Phaseolus vulgaris* cv. Derakhshan) leaves using the bacterial suspension from a 48-h old culture on YPGA medium at a concentration of 10^8^ CFU/mL (41). The pectinolytic activity was confirmed by the potato disks test, and the intensity of pectinolytic activity of the strains was assessed based on the intensity of rotting on the disks (42). All the tests were repeated twice. Based on the latter assays, a Gram-negative pectinolytic bacterial strain was isolated from the sporocarp of *S. luteus* in September 2021, while surveys in the subsequent year (September 2022) yielded another pectinolytic strain similar to that isolated in the previous year. Phenotypic features and biochemical characteristics of the strains were investigated using the procedure described by Schaad et al. (13). Details of the experimental procedure were described previously (43–45). In brief, Gram reaction, oxidase and catalase activities, aerobic/anaerobic growth, and colony characteristics on yeast extract-dextrose-calcium carbonate agar medium were determined. Enzymatic activity of the strains, hydrolyses of different substrates, and utilization of organic compounds were also investigated using the standard procedure (13). Based on the phenotypic characteristics of the strains, they were preliminarily identified as members of *Burkholderia* sp., while positive pectinase and HR activity suggested that they could possess phytopathogenic features. Thus, type/pathotype strains of *B. cepacia* CFBP 2227^T^, *B. gladioli* pv. *gladioli* CFBP 2427^PT^, and *B. gladioli* pv. *alliicola* CFBP 2422^PT^ and standard strain *B. cenocepacia* CFBP 8617 were used as controls in all the biochemical and phenotypic tests. All the tests were repeated twice.

### Pathogenicity test on edible mushroom

The strains Ir1503 and Ir1504 isolated from sporocarps of *S. luteus* under natural conditions could have had pathogenicity features on sporocarpous fungi including edible mushrooms. Thus, they were evaluated for their virulence on the sporocarps of white button mushrooms along with the reference strains of *Burkholderia* spp. and the mushroom pathogen *P. tolaasii* (46). Pathogenicity of the strains was evaluated (repeated twice) on the caps (sporocarps) of fresh white button mushrooms using the cut-cap method (9). In brief, the bacterial suspension was inoculated using a micropipette onto the cut surface of the caps (1 × 10^7^ CFU/mL in SDW; four spots/cap; 20 µL/spot). The inoculated specimens were incubated in a dark moist chamber at 24°C–27°C up to 72 hpi and periodically monitored for symptom development. Reference strains of *B. cepacia* CFBP 2227^T^, *B. gladioli* pv. *alliicola* CFBP 2422^PT^, *B. gladioli* pv. *gladioli* CFBP 2427^PT^, and *B. cenocepacia* CFBP 8617 were also used in the pathogenicity tests in the same manner. The brown blotch pathogen of mushroom *P. tolaasii* CFBP 8707 was used as a positive control, while the non-pathogenic strain of *E. coli* (dh5α) and SDW was used as negative controls. Koch’s postulates were accomplished by re-isolation and identification of bacterial strains from the symptomatic caps using colony morphology and Gram staining.

### Plant pathogenicity and host range assays

In order to decipher the pathogenicity and host range of the two mushroom-associated *Burkholderia* strains, they were inoculated on onion (the main host of *B. cepacia* and *B. gladioli* pv. *alliicola*) and gladiolus (the host of *B. gladioli* pv. *gladioli*) as well as taxonomically related species, that is, garlic (*Allium sativum*), narcissus (*Narcissus jonquilla*), spring onion (*Allium fistulosum*), and Persian shallot (*Allium stipitatum*). This assay would shed light not only on the pathogenicity features of the strains but also on their host range. Plant inoculation and virulence assessment were conducted according to the procedure described previously (44, 47). In brief, the vegetables and fruits were superficially disinfected with 1% sodium hypochlorite. Then, 10 µL of the bacterial suspension (10^7^ CFU/mL) was inoculated using a micropipette into the fleshy parts of these plants. The inoculated specimens were incubated in a moist chamber with a humidity of 80%–90% at 28°C for 72 hours.

Furthermore, the pathogenicity of the two strains was evaluated on the fleshy tissues of several vegetables and annual crops outside the established host range of phytopathogenic *Burkholderia* spp., that is, chili pepper (*Capsicum annuum; Solanaceae*), cucumber (*Cucumis sativus; Cucurbitaceae*), eggplant (*Solanum melongena; Solanaceae*), green bean (*P. vulgaris; Fabaceae*), kohlrabi (*Brassica oleracea; Brassicaceae*), mango (*Mangifera indica; Anacardiaceae*), okra (*Abelmoschus esculentus; Malvaceae*), potato (*Solanum tuberosum; Solanaceae*), and watermelon (*Citrullus lanatus; Cucurbitaceae*). The preparation of plant materials and inoculation procedure were the same as described above. Three replicates were used for each strain. Furthermore, *B. cepacia* CFBP 2227^T^, *B. cenocepacia* CFBP 8617, *B. gladioli* pv. *alliicola* CFBP 2422^PT^, and *B. gladioli* pv. *gladioli* CFBP 2427^PT^ were used as controls. The experiments were repeated twice.

### Phylogenetic analyses

Phenotypic features and colony characteristics suggested that the two strains had high similarity to *Burkholderia* spp. To decipher the exact taxonomic position of the strains, a phylogenetic analysis was conducted using the sequences of 16S rRNA along with *atpD*, *gyrB*, and *lepA* housekeeping genes (22). Sequences of the three housekeeping genes *atpD*, *gyrB*, and *lepA* have been shown to robustly delineate the phylogenetic position of different *Burkholderia* lineages (19, 22, 48). Bacterial DNAs were extracted using the Expin Combo GP (GeneAll) DNA extraction kit via a procedure recommended by the manufacturer. For PCR reactions, the Universal PCR Kit, AmpliqonTaq DNA Polymerase Master Mix Red (Ampliqon A/S, Odense, Denmark), was applied according to the manufacturer’s recommendations. For each strain, a 20 µL PCR including 50 ng of total DNA and 1 µL of each primer (10 pmol/µL) were used. The annealing temperatures and the corresponding primer sequences were described in the previous work (25). The PCR products were subjected to nucleotide sequencing via Sanger technology, and the resulting sequences were assembled, edited, and aligned using the combination of BioEdit, Clustal W, and MEGA7 software (49). The sequences of the three genes were concatenated in the alphabetic order of the genes, and phylogenetic trees were constructed using Maximum Likelihood method with 1,000 bootstrap replicates (50).

## Data Availability

The strains Ir1503 and Ir1504 isolated in this study are available in the French Collection for Plant-associated Bacteria (CFBP) and the International Collection of Microorganisms from Plants (ICMP) under the accession numbers lr1503 = CFBP 9103 = ICMP 24728 and lr1504 = ICMP 24720. Nucleotide sequences obtained in this study are deposited in the NCBI GenBank database under the following accession numbers OR263462-OR263463 for 16S rRNA, OR396931-OR396932 for *atpD,* OR424415-OR424416 for *gyrB,* and OR424417-OR424418 for *lepA*.

## SUPPLEMENTAL MATERIAL

Figures S1, S2, and S3 spectrum.03395-23-s0001.pdf Figure S1 (Pectinolytic activity of the strain Ir1503 on potato disk [A], and hypersensitive reaction of the same strain on tobacco [B] and common bean [C] leaves), Figure S2 (Inoculation of mushroom caps with non-pathogenic strain of *E. coli* dh5α [left] and brown blotch pathogen of mushroom *P. tolaasii* CFBP 8707 [right]; while brown blotch symptoms were observed on the mushroom caps inoculated with *P. tolaasii* CFBP 8707 [positive control], the caps inoculated with *E. coli* remained symptomless until 72 hours post inoculation), and Figure S3 (Pathogenicity of *Burkholderia gladioli* strains Ir1503 and Ir1504 on chili pepper).

ASM does not own the copyrights to Supplemental Material that may be linked to, or accessed through, an article. The authors have granted ASM a non-exclusive, world-wide license to publish the Supplemental Material files. Please contact the corresponding author directly for reuse.
