# Supplementary material for: Wild mushrooms as potential reservoirs of plant pathogenic bacteria: a case study on Burkholderia gladioli
Source: Microbiol Spectr. 2024 Feb 21;12(4):e03395-23. doi: 10.1128/spectrum.03395-23 (PMC10986547; doi:10.1128/spectrum.03395-23)
Supplement: Figures S1, S2, and S3 — Figure S1 (Pectinolytic activity of the strain Ir1503 on potato disk [A], and hypersensitive reaction of the same strain on tobacco [B] and common bean [C] leaves), Figure S2 (Inoculation of mushroom caps with non-pathogenic strain of E. coli dh5α [left] and brown blotch pathogen of mushroom P. tolaasii CFBP 8707 [right]; while brown blotch symptoms were observed on the mushroom caps inoculated with P. tolaasii CFBP 8707 [positive control], the caps inoculated with E. coli remained symptomless until 72 hours post inoculation), and Figure S3 (Pathogenicity of Burkholderia gladioli strains Ir1503 and Ir1504 on chili pepper). [file spectrum.03395-23-s0001.pdf]

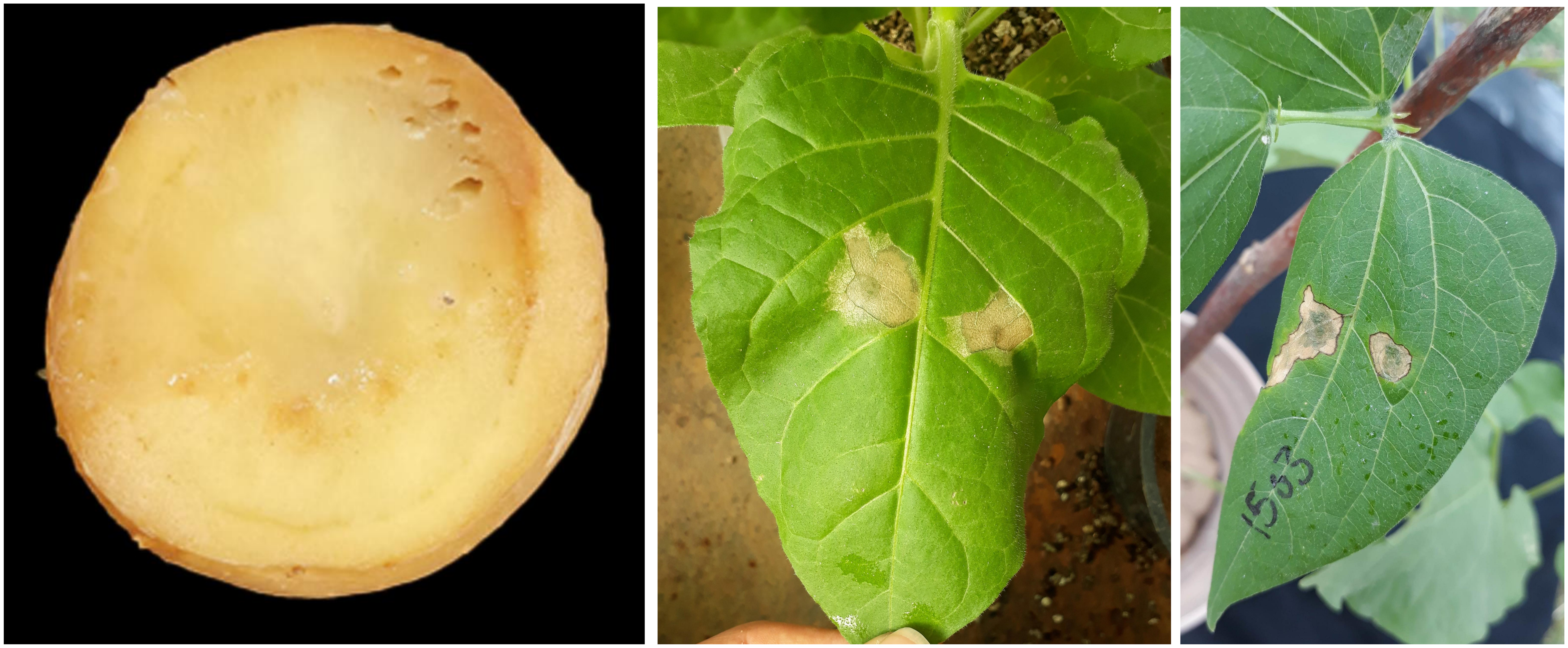

Figure S1: Pectinolytic activity of the strain IR1503 on potato disk (A), and hypersensitive reaction of the same strain on tobacco (B) and common bean (C) leaves.

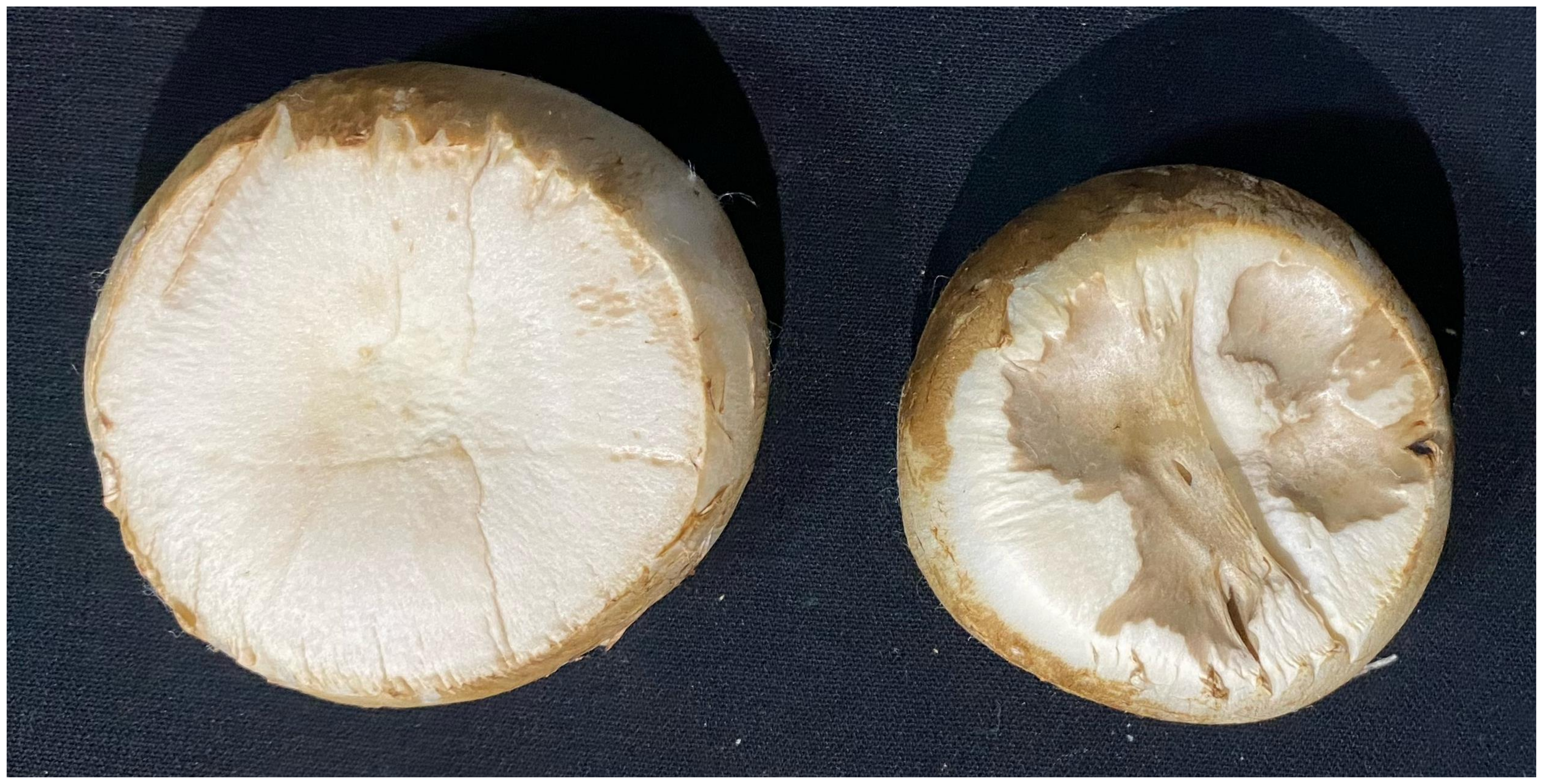

Figure S2: Inoculation of mushroom caps with non-pathogenic strain of Escherichia coli dh5α (left) and brown blotch pathogen of mushrooms *Pseudomonas tolaasii* CFBP 8707 (right). While brown blotch symptoms were observed on the mushroom caps inoculated with *Pseudomonas tolaasii* CFBP 8707 (positive control), the caps inoculated *E. coli* remained symptomless until 72 hours post inoculation.

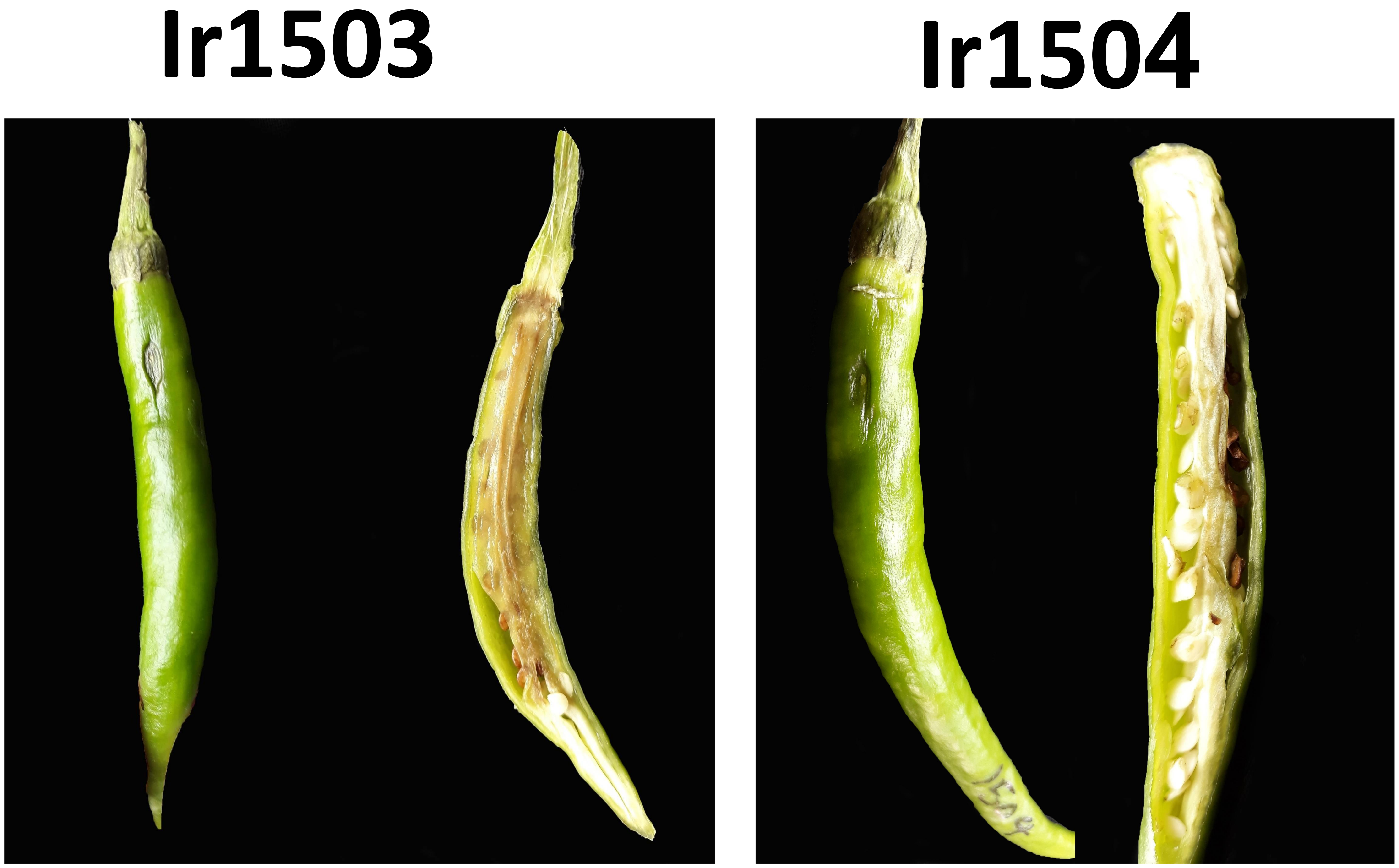

Figure S3: Pathogenicity of *Burkholderia gladioli* strains Ir1503 and Ir1504 on chili pepper.
